# 67 Does the volume or intensity of steps matter more on maximal oxygen consumption?

**DOI:** 10.1093/eurpub/ckae114.060

**Published:** 2024-09-26

**Authors:** Henri Vähä-Ypyä, Pauliina Husu, Kari Tokola, Harri Sievänen, Tommi Vasankari

**Affiliations:** UKK Institute, Tampere, Finland; UKK Institute, Tampere, Finland; UKK Institute, Tampere, Finland; UKK Institute, Tampere, Finland; UKK Institute, Tampere, Finland; Faculty of Medicine and Health Technology, Tampere University, Tampere, Finland

## Abstract

**Purpose:**

Both high step count and high intensity of steps are associated with better health outcomes. This study investigates how many daily steps or running steps are associated with higher maximal oxygen consumption (VO2max).

**Methods:**

The dataset was derived from 2135 female and 1439 male participants, aged 20-69 years, who took part in the FinFit2017- and FinFit2021population studies. Their VO2max was predicted using the 6-minute walking test, while daily step and running step counts were measured using accelerometers (UKK RM42). The female and male participants were categorized into ten groups based on VO2max deciles, weighed by age. Receiver operating characteristic (ROC) curves were used to determine the cut-points for the number of steps and running steps that were associated with a specified decile. The optimal cut-point was the point minimizing the Euclidean distance between the ROC curve and the (0,1) point.

**Results:**

The area under curve (AUC) for steps decreased from 0.739 to 0.642 in females and from 0.709 to 0.597 in males as the VO2max decile increased. At the lowest decile, the optimal cut-point was 6033 steps per day for females and 5474 steps per day for males. The step cut-point plateaued at the VO2max level of 30 ml/kg/min for females and at the VO2max level of 35 ml/kg/min for males.

The AUC and cut-points for the running steps showed an upward trend with increasing VO2max. AUC values ranged from 0.686 to 0.788 for females and from 0.701 to 0.768 for males. The cut-points at the highest decile were 234 running steps per day for females and 238 for males.

**Conclusions:**

In this cross-sectional sample of working-aged adults, a greater number of daily steps was associated with higher VO2max up to 7000 steps per day, whereas the running steps did not show a plateau effect. Consequently, our results suggest that individuals with lower levels of physical activity can benefit from increasing their overall physical activity volume. On the other hand, those having higher volumes of physical activity may benefit more by increasing the intensity of their physical activity.

**Support/Funding Source:**

Finnish Ministry of Education and Culture

